# Self-management of psoriasis vulgaris treatment burden: A Review

**DOI:** 10.1097/MD.0000000000035392

**Published:** 2023-11-03

**Authors:** Dan Zhu, Na Li, Xiang Yan, Meng Zheng

**Affiliations:** a Department of Dermatology, Kunming Medical University First Affiliated Hospital, Kunming, China.

**Keywords:** best evidence, psoriasis vulgaris, self-management, treatment burden

## Abstract

Psoriasis vulgaris is complicated with metabolic syndrome and other diseases, which affects the longevity of patients. Its repeated attacks bring obvious and long-term treatment burden to patients, and improper self-management can aggravate disease symptoms and increase the risk of complications. To summarize the existing evidence on the self-management of psoriasis vulgaris treatment burden. Systematic search was performed in database. Two researchers reviewed, extracted, and summarized evidence from the literature. Nine studies were included, of which 3 guidelines, the number of fields with standardized scores in various domains ≥60% of the evaluation results of the 3 guidelines was at least 4, quality evaluation are all above Grade B, demonstrated higher quality of guidelines, 3 RCTs, 2 analytical studies, and 2 literature reviews. The evaluation results of these included literatures were of high quality. We summarized the data in 6 areas, including oral medication management, topical treatment management, risk factors, living habits, physical exercise, and biologics managements. Finally, we present 34 items of best evidence. This study provides the basis for the management of treatment burden of psoriasis vulgaris. According to this study, medical workers guide patient to reduce the disease and treatment burden.

## 1. Introduction

Psoriasis vulgaris and metabolic syndrome are chronic systemic inflammatory diseases,^[1–3]^ with a close association between them. Patients with psoriasis vulgaris are at an increased risk of developing metabolic syndrome and its associated diseases and vice versa.^[4,5]^ Treatment burden refers to the physical and psychological impact of medical care workload on patients.^[6]^ It includes the care and self-management activities that patients must perform and the physical, psychological, behavioral, and cognitive impact of these activities.^[7]^ As a recurrent disease, psoriasis vulgaris imposes a significant and long-term treatment burden on patients, and improper management can exacerbate disease symptoms and increase the risk of comorbidities^[7]^ Treatment burden in patients with psoriasis vulgaris has attracted considerable research attention, but no relevant high-quality evidence has been published. Therefore, it is important to summarize the best evidence on the self-management of treatment burden in patients with psoriasis vulgaris to provide a basis for its introduction into the clinical practice of healthcare professional.

## 2. Methods

### 2.1. Research question identification

The research question of this study was to determine which measures could effectively promote improved self-management in patients with psoriasis vulgaris, leading to an outcome of reduced treatment burden. To obtain the best evidence, we selected the initial questions for this evidence-based care using the PICOT approach, namely P (population): age ≥ 18 years, diagnosis of psoriasis vulgaris; I (intervention): improved self-management ability; C (comparison): current patient self-management ability; O (outcome): improved patient ability to self-manage disease and reduced treatment burden; T (type of evidence): guidelines, evidence summaries, expert consensus, systematic reviews, and randomized controlled trials.

### 2.2. Evidence retrieval strategy

A computerized evidence search was conducted from database creation to March 31, 2022, based on the “6S” evidence model.^[8]^ The databases that we searched for evidence were the following: The National Network of Guidelines (NGC), China Clinical Guidelines Collection (CGC), JBI Evidence-Based Health Care International Collaborating Center Library, National Health Care Commission of the People Republic of China (formerly Ministry of Health), China Psoriasis Network (CPC), American Academy of Dermatology (AAD), CHKI, Wanfang, PubMed, Web of Sciences, Scopus, and Embase. The Chinese language database search terms were as follows: “psoriasis/treatment burden/self-management/continuing care/symptom management/community care/chronic disease management.” The English database search terms were the following: “Psoriasis Vulgaris/Psoriasis/Treatment Burden/ Continuing care/Symptom management/Self-management/Community nursing/Chronic disease management.” The search term was expanded to include “treatment/burden/care/psoriasis/skin lesion management/disease management/chronic inflammation/symptom burden” due to the small number of results. The search results are still the same.

### 2.3. Literature inclusion and exclusion criteria

The literature inclusion criteria were as follows: literature and guidelines involving the burden of psoriasis vulgaris treatment, self-management of patients with psoriasis vulgaris, and management of chronic disease; language range: English and Chinese; time period: from the establishment of the database to present time. The following exclusion criteria were applied: incomplete data; duplicate literature. The criteria for deletion were the following: not relevant to the topic of psoriasis vulgaris and not in line with the current general nursing practice. Two researchers integrated and extracted the content of the included literature.

### 2.4. Criteria for literature quality evaluation

The Clinical Guidelines Research and Evaluation System^[8]^ was used to evaluate the included literature. Each type of study was evaluated separately using the Australian JBI Centre for Evidence-Based Health Care quality evaluation tool.^[9–12]^

### 2.5. Evidence quality evaluation

Evidence extraction was done independently by 3 investigators after systematic evidence-based training. Conflicting evaluation opinions between 2 investigators were resolved through discussion. In cases, in which the determination for inclusion or exclusion was difficult, subject matter experts made the decisions based on the principles of evidence-based care. The inclusion principle followed in this study was the preference for high-quality evidence with the principles of evidence-based medicine.

## 3. Results

### 3.1. General characteristics of the included literature

**Figure 1.**
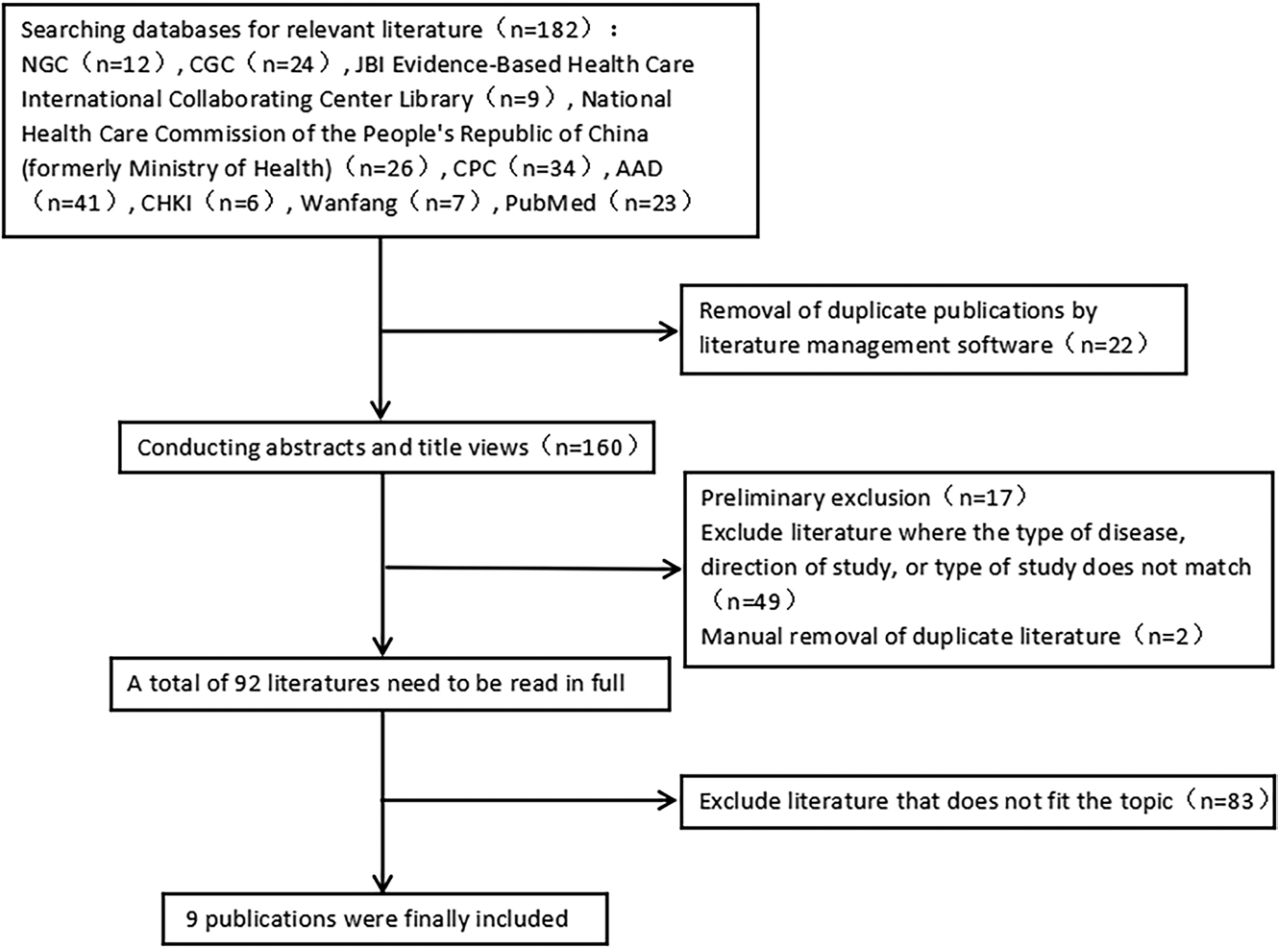
Flow chart for literature screening.

Based on the search results, 238 publications were initially included. The literature evaluation process is illustrated in Figure 1. After the nadir criteria, 9 publications were finally included: 3 guidelines,^[13–15]^ 2 analytical studies,^[16,17]^ 3 RCTs,^[18–20]^ and 1 literature review^[21]^ (Table 1).

**Table 1:** Characteristics of the included studies.

Author	Literature source	Evidence type	Literature content/theme	Publication time
Nast A. et al^[13]^	PubMed	Guideline	Psoriasis vulgaris treatment goals and treatment recommendations	2021
Nast A. et al^[14]^	J Eur Acad Dermatol Venereol	Guideline	systemic treatment of psoriasis vulgaris	2017
Julia-Tatjana Maulet al^[15]^	PubMed	Guideline	Topical treatment of psoriasis vulgaris	2021
Xu et al^[16]^	CNKI	Analytical studies	Relationship between patient self-management typing and quality of life by cluster analysis	2021
Zhang et al^[17]^	CNKI	Analytical studies	Effect of Sequential Therapy Adherence on Condition and Quality of Life	2020
Lou et al^[18]^	CNKI	RCT	Analysis of factors related to disease recurrence and self-management intervention study	2021
Zhang et al^[19]^	CNKI	RCT	Health Belief Model Intervention Body Image Barriers and self-management skills	2020
Wan et al^[20]^	CNKI	RCT	Health belief model interventions for quality of life and ability to self-manage illness	2020
Ying et al^[21]^	CNKI	Review	Metabolic syndrome with psoriasis	2021

### 3.2. Quality evaluation results of the included studies

#### 3.2.1. Quality evaluation results of the guideline.

Three guidelines,^[13–15]^ was included in this study, and the percentage of standardization in each field is presented in Table 2. The study design was complete, and the overall quality was high.

**Table 2:** Methodological quality evaluation of the guidelines.

Study	Standardized scores in various domains (%)						≥60%	≥30%	Quality evaluation
	Scope and purpose	Stakeholder involvement	Rigor of development	Clarity of presentation	Applicability	Editorial independence			
Nast A. et al^[13]^	83.33%	66.67%	53.33%	81.67%	27.83%	70%	4	5	B
Nast A. et al^[14]^	70.05%	70.58%	73.77%	75.80%	29.13%	83.48%	5	5	B
Julia-Tatjana Maulet al^[15]^	77.54%	85.36%	79.21%	81.1%	66.52%	69.87%	6	6	A

#### 3.2.2. Quality evaluation results of analytical studies.

A total number of 2 analytical studies were included in this study,^[16,17]^ (Table 3).

**Table 3:** Methodological quality evaluation of the analytical studies.

Evaluation criteria	Xu et al^[16]^	Zhang et al^[16]^
1. For the study, is the researcher clear about what he or she knows and what he or she does not know? How does the research address the research gaps?	Yes	Yes
2. Is the purpose of the study clear?	Yes	Yes
3. Is the literature review recent (most sources are within the last 5 yr or traditional)?	Yes	Yes
4. Is the sample size based on the study design and the underlying rationale adequate?	No	Unclear
5. Are the characteristics and/or statistics similar in the control and intervention groups?	Unclear	Unclear
6. If multiple backgrounds of research are used, are the backgrounds similar?	Unclear	Yes
7. Are all groups treated equally, except for the intervention group?	Unclear	Yes
8. Does it clearly describe how the data was collected?	Yes	Yes
9. Are research tools reliable? (Cronbach′s α ≥ 0.70)	Unclear	Unclear
10. Has the effectiveness of the research tools been discussed?	Unclear	Unclear
11. If questionnaire is used, is the response rate ≥ 25%?	Unclear	Unclear
12. Are the results clearly presented?	Yes	Yes
13. If a table is used to describe the results, is the description consistent with the table content?	Yes	Yes
14. 14 Were there limitations of the study identified and addressed?	Yes	Yes
15. Are the conclusions based on results?	Yes	Yes

#### 3.2.3. Quality evaluation results of the randomized controlled trial.

A total number of 3 randomized controlled trials were included in this study^[18–20]^ (Table 4).

**Table 4:** Methodological quality evaluation of the randomized controlled trials.

Evaluation criteria	Lou et al^[16]^	Zhang et al^[19]^	Wan et al^[20]^
1. Was random grouping actually used for the study subjects?	Yes	Yes	Yes
2. Is the distribution hidden?	Yes	Yes	Yes
3. Is the baseline comparable between groups?	Yes	Yes	Yes
4. Were the study subjects blinded?	Yes	Unclear	Yes
5. Was the interventionist blinded?	Unclear	Unclear	Yes
6. Were the results blinded to the evaluator?	Yes	Yes	No
7. Did the groups receive the same measures other than the interventions to be validated?	Yes	Yes	Yes
8. Was the follow-up period completed? If not, were measures taken to deal with the loss of follow-up?	Unclear	Unclear	Unclear
9. Were all study subjects randomly assigned and included in the outcome analysis?	Yes	Yes	Yes
10. Were the outcome indicators measured in the same way for each study group?	Yes	Yes	Yes
11. Are the measures of outcome indicators credible?	Yes	Yes	Yes
12. Is the data analysis method appropriate?	Yes	Yes	Yes
13. Is the study design reasonable? Were there differences in the implementation of the study and in the analysis of the data from a standard randomized controlled trial?	Yes	Yes	Yes

### 3.3. Results of the quality evaluation of the literature review

One literature review was included in this study^[21]^ (Table 5).

**Table 5:** Methodological quality evaluation of the literature review.

Evaluation criteria	Ying et al^[21]^
1. Is the stated topic clearly stated?	Yes
2. Does the review contain relevant, up-to-date literature (most of the literature is within 5 yr or is of high quality)?	Yes
3. Does the review contain a meaningful analysis of the conclusions?	Yes
4. Does it describe the limitations in the review?	Unclear
5. Were the recommendations for future practice or research reached?	Yes

### 3.4. Evidence summary and analysis

Through the evaluation and integration of the evidence, 34 best evidence points were summarized in 6 aspects, including oral medication management, topical treatment management, risk factors, living habits, physical exercise, and biologics managements (Table 6).

**Table 6:** Evidence summary for psoriasis vulgaris treatment burden self—management.

Subject of evidence	Evidence content	Evidence level	Recommendation
Oral medication management	1. The capsules can be taken with a meal containing some fat or with whole milk to improve absorption.^[13]^	**5b**	**B**
	2. Ciclosporin (CsA)^[13]^:	**5c**	**A**
	During therapy with low-dose ciclosporin (CsA; 2.5 to 3 mg/kg body weight daily), follow-up intervals may be extended to 2 months or more with daily blood pressure monitoring. The follow-up intervals in patients with risk factors, were shortened after the dose increases, or for patients with drugs that may cause adverse drug reactions. Nursing condition observation/follow-up points should focus on skin and mucous membrane status, infection, gastrointestinal or neurological symptoms, and muscle/joint pain. Follow-up examinations after discontinuation should be performed about skin cancer, especially in high cumulative doses of previous drugs or with natural UV exposure areas (Yunnan).		
	3. Acitretin^[13]^:	**5b**	**A**
	Blood donation is not allowed during treatment and for 3 yr after cessation of treatment Drugs have teratogenic risks and require effective long-term contraception (3 yr after stopping treatment) Maintain abstinence from alcohol and a low-fat, low-carbohydrate diet to prevent elevated blood lipids and liver enzymes		
	4. Medication regimens may take appropriate account of patients’ personal preferences and regularly assess their satisfaction with treatment and outcomes^[15]^	**5b**	**A**
Topical treatment management	5. The use of emollients restores skin barrier function and prevents or reduces the frequency/extent of psoriasis vulgaris^[15]^	**5c**	**A**
	6. Patients are to be clearly informed of the teratogenic risks of the drug, the need for effective long-term contraception (3 yr after treatment termination) ^[15]^	**5c**	**A**
	7. Emollients are to be applied twice daily (morning and evening), more often as needed^[15]^	**3c**	**A**
	8. Ointments, creams or gels, lotions for very, moderately, and mildly dry skin respectively^[15]^	**5c**	**A**
	9. Patients are advised to pay attention to gentle wiping, appropriate water temperature, not too long when bathing, and apply emollients immediately after drying^[14,15]^	**5c**	**A**
	10. Recommended weekly dosage of emollient creams and ointments: 15–30 g for face, 25–50 g for hands, 50–100 g for scalp, 100–200 g for upper and lower extremities, 400 g for the trunk, 15–25 g for the groin and genitals, depending on the degree of dryness of the skin, 20 g/time for mild, 50 g/time for moderate, and 100 g/time for severe conditions (for scalp/perineum area, it is recommended when rubbing topical medication (skin preparation), evaluation of the dosage: the size of the patient one fingertip (the first knuckle of the index finger) can be applied to both hands, each time to take the amount of a fingertip, for particularly dry skin can be increased as needed (two fingertips)^[15]^	**1c**	**A**
Risk factors	11. The purpose of using non-hormonal creams alone during the disease control phase is to control the disease condition^[15]^	**3c**	**B**
	12. Patients with psoriasis vulgaris are advised to regularly monitor blood biochemical indicators during drug therapy to prevent cardiovascular disease^[14,18]^	**5c**	**A**
	13. Patients are asked to quit smoking^[17]^	**1b**	**A**
	14. In winter and spring, patients are advised to increase the use of emollients^[17,18]^	**3c**	**A**
	15. Patients should keep a good mood and get sufficient sleep^[14]^		
	16. Patients are required to seek medical care to avoid delaying of the disease process treatment^[17]^	**5b** **5c**	**A** **A**
	17. Alcohol affects immune function, patients should limit alcohol intake^[17]^		
	18. Psoriasis patients should consistently self-manage^[16]^	**5c**	**B**
	19. Regular follow-up can enhance patient compliance^[17]^		
Living habits	20. Psoriasis vulgaris is a systemic disease that requires lifestyle changes, including a healthy diet, regular exercise, and smoking cessation, as well as control of high blood pressure and regulation of blood lipids^[16]^	**5b** **5b**	**B** **A**
	21. Health education and psychological intervention can help control the disease^[16]^	**5c**	**A**
	22. Psoriasis patients should avoid high-fat diets to prevent comorbidities^[14]^		
	23. Early detection and management of cardiovascular disease risk factors, timely and effective control of the disease, rational use of medication, and active lifestyle adjustment can reduce the risk of cardiovascular disease in combination with psoriasis to a certain extent^[17–19]^	**5c** **1c**	**A** **A**
	24. The needs of patients are documented, their reasonableness is assessed, and meeting them is attempted^[16]^	**3c**	**A**
	25. Excessive exposure to the sun is to be avoided, physical sun protection measures are to be implemented, such as wearing hats, sunglasses, and masks (In areas of high UV light)^[14,15]^	**3c**	**B**
	26. Exercise helps prevent chronic disease and improve mental health by incorporating physical activity into intervention programs to increase well-being and reduce negative emotional states^[19,20]^	**1c**	**A**
Physical exercise	27. The average rate of lesions was lower in patients who exercised regularly than in those who exercised less^[17]^	**1c** **1c**	**A** **B**
	28. After the physical fitness assessment, the patient exercise prescription is formulated to meet his or her needs and to assist in systemic treatment^[13]^	**5c**	**A**
	29. Physical activity 4 or more times a week is to be encouraged, and the patient wishes need to be considered^[14]^	**5c**	**A**
	30. Check whether the patient has malignant tumor, active tuberculosis, infection, liver and kidney function, history of previous treatment, and heart function before using biologics^[13]^	**3c**	**A**
Biologics managements	31. During biologic treatment, the focus is on infection, and the presence of malignant tumors, especially skin cancer, precancerous lesions, congestive heart failure and neurological symptoms, and the presence of allergies^[13]^	**5c**	**A**
	32. Strict contraception during biologic treatment^[13]^	**5b**	**A**
	33. Regular monitoring of blood pressure, liver and kidney function during biologic therapy^[13]^	**5c**	**B**
	34. Appropriate tests and discontinuation of contraception (if required) after completion of biologic treatment^[13]^		

## 4. Discussion

This study was focused on 6 relevant areas in patients with psoriasis vulgaris, including oral medication management, local treatment management, risk factors, lifestyle habits, physical activity, and biologic agent management. In the course of clinical practice, compliance with oral medication in patients with psoriasis vulgaris is one of the most important factors affecting the control of the condition and promoting the recovery of the skin lesions.^[22]^ The goal of clinical therapeutic care management is not only to determine the treatment regimen, but to focus on the implementation of the regimen. Including the manner of medication administration and management of drug side effects and monitoring of post-medication changes, and special medication precautions. Evidence from 1 to 6 present commonly used drugs, the methods of medication administration, and the points of observation after medication administration. Medical practitioners educate patients how to administer medication and assess disease progression, and how to observe the medication side effects. The progression of disease is a negative aspect of treatment burden, and after medical staff teach patients to properly understand it, self-management of treatment burden can be promoted. Additionally, patients can improve their learning about the disease and obtain related knowledge.^[23]^

Topical drug therapy is an important treatment modality in psoriasis treatment, and patients with psoriasis are often unclear about the dosage and method of topical drug administration. The correct dosage and method can greatly improve the therapeutic effect of drugs on the lesions and reduce drug wastage. Evidence 7 to 11 indicate the specific dose usage and need for topical medications in patients with psoriasis vulgaris. Skin lesions are important factor that aggravates the treatment burden, and thus their removal is an urgent requirement. Skin lesions cause a further deterioration of quality of life, including that of physical, mental, and social health status. Nevertheless, it can have a positive side regarding its educational role and behavioral improvement in patients that can promote their health self-management. Medical practitioners should actively train patients in applying correctly drugs topically by rubbing and teach them to use more effective methods and approaches for the treatment of different skin lesions as well as medications to promote their recovery. For example, important points are how to calculate the precise dosage of topical medications and how to choose the massage technique to be applied, as well as the sealing packs and wet compresses to promote the absorption and efficacy of topical medications.

There are various triggers for psoriasis flare-ups and a key point to achieve long-term disease control to reduce flare-ups is to identify and prevent the effects of risk factors. Evidence 12 to 16 identify risk factors to be aware of, for example, smoking and alcohol. Clinical personnel to instruct patients on how to reduce risk factor exposure to decrease the probability of disease recurrence and diminish the treatment burden on patients. By reducing the patient exposure to risk factors for recurrence or exacerbation of the disease. For patients can reduce the pain caused by the disease and duplication of see the doctor, but also reduce the pressure of medical institutions to receive treatment, promote the disease process of self-management, reduce the degree of treatment burden.

Poor lifestyle habits can lead to increased lesions and exacerbation of the disease and a reduced quality of life. Evidence 17 to 25 present aspects in the lifestyle habits of patients with psoriasis that patients need to be aware of in their daily lives. Patients can improve disease management outcomes by improving their lifestyle habits. Self-management should be integrated throughout the treatment of psoriasis vulgaris.^[1,22]^ Maintaining healthy lifestyle habits is not only beneficial to the control of disease symptoms, but also to maintaining health over time and reducing disease flare-ups. Patient management of lifestyle habits may add to the burden of the patient personal, family, community, and professional roles. The contradiction between the purpose and modality of lifestyle habit management leads to and influences the creation and change of treatment burden. If the contradiction is not taken seriously and dealt with, it can affect and reduce the treatment effect, or even lead to disease aggravation, forming a vicious circle and increasing the risk of doctor-patient conflict, finally affecting the whole treatment effect. Clinicians should conduct a comprehensive assessment together with the patient and jointly develop a reasonable treatment plan to achieve a better treatment effect and reduce the treatment burden.^[24]^

Psoriasis vulgaris is pathologically characterized by a high degree of vascular origin and is manifested by proliferation of vascular endothelial cells, dilated capillaries, and increased permeability. Physical exercise increases the elasticity of the blood vessel walls, which is very beneficial for the long-term outcome of healthy disease management in psoriasis patients A negative correlation between activity intensity and psoriasis severity has been found, but there is a lack of professional physical activity instruction for patients, and the assessment methods need to be improved.^[25]^

Evidence 26 to 29 specify the methods and precautions for physical activity in psoriasis, and clinicians guide patients to perform appropriate physical activity according to their condition. Future studies can be performed in combination with sports specialties to develop specifically applicable exercise methods for patients with psoriasis vulgaris that can assist in the treatment and can contribute to symptoms improvement.

Biological agents can provide precise inhibition of specific targets and are widely used in clinical practice. Most biologics have been approved by state authorities and have been included in medical insurance, and patients are willing to undergo biologic agent therapy. As biologics have become one of the mainstream treatment modalities for psoriasis vulgaris, it is important to have scientific evidence to guide medical staff in areas that need to be monitored and focus on before, during, and after patient treatment. Evidence 30 to 34 describe aspects of biologic therapy that are relevant. Minimizing the side effects associated with biologics administration and relieving patient physical and psychological stress are also necessary to reduce the patient treatment burden.^[26]^ Since patients receive biologic injections mostly on a regular basis in outpatient clinics and are not hospitalized, medical staff should teach patients to actively experience changes in their condition after a medication has been administered and regular review, which is also a part of treatment burden management.^[6,27]^

## Author contribution

**Conceptualization:** Dan Zhu, Na Li, Meng Zheng.

**Data curation:** Dan Zhu, Na Li, Xiang Yan, Meng Zheng.

**Formal analysis:** Dan Zhu, Na Li, Xiang Yan.

**Funding acquisition:** Xiang Yan.

**Investigation:** Dan Zhu.

**Methodology:** Xiang Yan, Meng Zheng.

**Project administration:** Xiang Yan.

**Resources:** Meng Zheng.

**Writing – original draft:** Dan Zhu, Na Li, Xiang Yan, Meng Zheng.

**Writing – review & editing:** Dan Zhu, Na Li, Xiang Yan, Meng Zheng.
